# Thank you to reviewers

**DOI:** 10.1093/eurpub/ckaf006

**Published:** 2025-02-17

**Authors:** Peter Allebeck, Diana Delnoij, Thomas Dorner, Chiara de Waure

A scientific journal exists because of the good will and assistance of its reviewers, who generously contribute their time and knowledge. As a journal, we can only publish approximately 25% of the papers we receive. In order to publish high-quality papers, we depend on the expertise and recommendations of the reviewers. In 2024, a total of 423 reviewers provided their expert opinions to guide our editorial decisions. We would like to take this opportunity to thank each of our reviewers, as listed below:

Adawi Mohsen Abdu, Saudi Arabia

Enrique Acosta, Spain

Tim Adair, Australia

Charles Agyemang, Netherlands

Saeed Akhtar, Kuwait

Seif Alabri, Oman

Barrak Alahmad, United States

Lubna Al-Ariqi, Saudi Arabia

Tit Albreht, Slovenia

Gedefaw Alen, Australia

Hamzeh Alipour, United States

François Alla, France

Melody Almroth, Sweden

Carlotta Amantea, United States

Ingelise Andersen, Denmark

Nick Andrews, United Kingdom

Thomas Anker, United Kingdom

Cigdem Apaydin Kaya, Turkey

Franklin Apfel, United Kingdom

Bénédicte Apouey, France

Ramiz Arabaci, Turkey

Emilia Aragon de Leon, Denmark

Fatime Arenliu Qosaj, Albania

Lucia Artazcoz, Spain

Rahel Asefa, United States

Asokan Asokan GV, Bahrain

Giovanni Aulino, Italy

Philippe Autier, France

Eileen Avery, United States

Ileana Baldi, Italy

Birna Baldursdottir, Iceland

Zakaria Bameri, United States

Helen Banks, Italy

Devendra Bansal, Qatar

Amrit Banstola, United Kingdom

Tom Baranowski, United States

José Augusto Barreto Filho, Brazil

Arne Bathke, Austria

Latifa Baynouna AlKetbi, United Arab Emirates

Julien Beauté, Sweden

Flavia Beccia, Italy

Jasper Been, Netherlands

Deepak Kumar Behera, Viet Nam

Ana Belmonte, Spain

Sherihane Bensemmane, Belgium

Carla Berg, United States

Anke Bergmann, Germany

Merita Berisha, Albania

Praveen Kumar Bharti, United States

Prerna Bhasin, India

Alison Bish, United Kingdom

Jonas Björk, Sweden

Ingunn Bjornsdottir, Denmark

Mary Black, United Kingdom

Vendula Blaya-Nováková, Spain

Jenni Blomgren, Finland

Dannel Boatman, United States

Aurélie Bocquier, France

Claire Boone, Canada

Karen Maria Borg, Malta

Rikke Borg, Denmark

Lisa Bornscheuer, Sweden

Isabelle Bos, Netherlands

Guillem Bosch Duran, United States

Marwân-al-Qays Bousmah, France

Maria Piedade Brandão, Portugal

Joao Breda, Greece

Elizabeth Breeze, United Kingdom

Sven Bremberg, Sweden

Giovanni Leonardo Briganti, Italy

Oscar Brück, Finland

Laura Brunelli, Italy

Silvio Brusaferro, Italy

Duncan Buchanan, United States

Alex Burdorf, Netherlands

Howard Burkom, United States

Bo Burström, Sweden

Stefan Buttigieg, Malta

Stephan Buttigieg, United States

Chiara Cadeddu, Netherlands

Tomaž Čakš, Slovenia

Alec Cali, Netherlands

Sharon Campbell, United States

Pablo Campos-Garzon, Spain

Margarida Cardoso, Portugal

Francesco Andrea Causio, Italy

Andrea Causio, Italy

Yue-Fang Chang, United States

Mafaten Chaouali, United States

Janine Charnley, Australia

Elisabetta Chellini, Italy

Kong-Tong Chen, Taiwan

Geneviève Chêne, France

Nain-Feng Chu, Taiwan

Tom Clemens, United Kingdom of Great Britain and Northern Ireland

Sheelah Connolly, Ireland

Claudia Costa, Portugal

Peter Croft, United Kingdom

Paolo Crosignani, Italy

Bruno da Costa, Brazil

Shaonong Dang, China

Mike Daube, Australia

Asli Davas, Turkey

Pauline de Heer, Netherlands

Harry de Koning, Netherlands

Elise de La Rochebrochard, France

Augusto Cesar De Moraes, Brazil

Bertrand de Neuville, France

Corrado De Vito, Italy

Chiara de Waure, Italy

Ulrik Deding, Denmark

DJH Deeg, Netherlands

Claire Demiot, France

Evangelia Demou, United Kingdom

Sarah Denford, United Kingdom

Alina Denham, United States

Sevgi Deniz Doğan, Turkey

Alexis Descatha, France

Annabel Desgrées du Loû, France

Marcello Di Pumpo, Italy

Julio Diaz, Spain

Finn Diderichsen, Denmark

Henriëtte Dijkshoorn, Netherlands

Dragana Dimitrijevic, United States

Annette J Dobson, Australia

David Doku, Ghana

Laia Domingo, Spain

Felıcitas Domınguez-Berjon, Spain

Anne Doyle, Ireland

Julie Dreier, Denmark

Thomas Drivsholm, Denmark

Frida Eek, Sweden

Rabie El Arab, Saudi Arabia

Habida Elachola, United States

Marie Eliasen, Denmark

Mubarak ELkarsani, United States

Joost Enzing, Netherlands

Saffet Erdoğan, Turkey

Erdem Erkoyun, Turkey

Bernabe Escober Perez, Spain

Olivier Ethgen, Belgium

Fabrice Etile, France

Petra Fadgyas-Freyler PhD, Hungary

Eliane Faria, Brazil

Sara Farina, Italy

Christina Fastl, Austria

Daniel Feikin, Switzerland

Nihad Fejzic, Bosnia and Herzegovina

Gianluigi Ferrante, Italy

Tommaso Filippini, Italy

Carmine Finelli, Italy

Patricia Fitzpatrick, Ireland

Peter Flach, Netherlands

Anders Foldspang, Denmark

Valter Fonseca, Greece

Joan Font, United Kingdom

Leena Forma, Finland

Stefan Fors, Sweden

Rosemary Frasso, United States

Judith Fuchs, Germany

Florentina Furtunescu, Romania

Gauden Galea, Denmark

Artur Galimov, United States

Mandy Geise, Netherlands

Laurent Gerbaud, France

Krisztina Gero, United States

Ramy Ghazy, Saudi Arabia

Alejandro Gil-Salmerón, Spain

Thordis Gisladottir, Iceland

Rosa Gofin, Israel

Léontine Goldzahl, France

Esther Golnabi, United States

Evangelia Grammatikaki, Greece

Scott Greer, United States

Pavel Grigoriev, Germany

Matthew Groenewold, United States

Wei Gu, Luxembourg

Montserrat Guillen, Spain

Faisal Hafeez, Pakistan

Carita Håkansson, Sweden

Kai Haldre, Estonia

Anne Hammarström, Sweden

Huiju Han, China

Mark Hann, United Kingdom

Guang Hao, China

Rannveig Hart, Norway

Jieyu He, China

Jenny Head, United Kingdom

Gunnel Hensing, Sweden

Lucinda Hiam, United Kingdom

Lenka Hodacova, Czech Republic

Theodore Holford, United States

Anna-Clara Hollander, Sweden

Bjorn Holstein, Denmark

Mark Horowitz, United Kingdom

Hanno Hoven, Germany

Candice Howarth, United Kingdom

Amanda Howe, United Kingdom

David Hu, United States

Cheng-Yang Hu, China

Lihong Hu, United States

Hai Hu, China

Caroline Huas, France

Juliette Hussey, Ireland

Katherine Ianni, United States

Ivo Iavicoli, Italy

Isabel Iguacel, Spain

David Ingleby, Netherlands

Paul Isenman, United States

Georgi Iskrov, Bulgaria

Josephine Jackisch, Switzerland

Kinga Janik-Koncewicz, Poland

Urban Janlert, Sweden

Staffan Janson, Sweden

Asko Järvinen, Finland

Domantas Jasilionis, Germany

Magnus Johansson, Sweden

Olalekan John, United States

Karsten Jørgensen, Denmark

Heather Joshi, United Kingdom

Mojca Juricic, Slovenia

Marja Jylhä, Finland

M Kakizaki, Japan

James Kang, United Kingdom

Ghazi kayali, United Arab Emirates

Lital Keinan-Boker, Israel

Myriam Khlat, France

Katarzyna Kissimova-Skarbek, Poland

Marte Kjøllesdal, Norway

Chiori Kodama, Egypt

Barbara Kondilis, Greece

Kaarina Korhonen, Finland

Zsigmond Kosa, United States

ipek köse tosunöz, United States

Shafi koya, Egypt

Gloria Krahn, United States

Lindy Kregting, Netherlands

Alfgeir Kristjansson, Iceland

Thomas Kühlein, Germany

Tilman Kühn, Austria

Christina N Kyriakos, United Kingdom

Carlo La Vecchia, Italy

Carina Ladeira, Portugal

Taavi Lai, Estonia

Outi Lapatto-Reiniluoto, Finland

Elin Larsson, Sweden

Taina Leinonen, Finland

Karina Leksy, Poland

Giovanni Leonardi, United Kingdom

Tinne Lernout, Belgium

Rüdiger Leutgeb, Germany

Yi Liang, China

Ulrik Lidwall, Sweden

Pelle Lindqvist, United States

Yihai Liu, China

Yan Liu, Japan

Daniel Lüdecke, Germany

Birca Ludmila, Moldova

Julia Lynch, United States

Heidi Lyshol, Norway

Morag MacKay, United States

David Madden, Ireland

Els Maeckelberghe, Netherlands

João Paulo Magalhães, Portugal

Eman Mahfouz, Egypt

Barbara Mahon, United States

Vittorio Maio, United States

Elfatih Malik, United States

Sierk Marbus, Netherlands

Otilia Mårdh, Sweden

Marco Mariani, Italy

Leslie Martin, United States

Homero Martínez, Canada

Miguel Martín-Matillas, Spain

Edgar Maydana, Spain

David McKee, United Kingdom

Sally McManus, United Kingdom

Enkeleint Aggelos Mechili, Albania

Maria Melchior, France

Inés Mesa Eguiagaray, United Kingdom

Masanari Minamitani, Japan

Anu Molarius, Sweden

Joyce Molenaar, Netherlands

Graham Moore, United Kingdom

Ian Morgan, Australia

Damon Morris, United Kingdom

Hanns Moshammer, Austria

Maria Mourão-Carvalhal, Portugal

Antons Mozalevskis, Denmark

Yuichi MURAKI, Japan

Mario Muselli, Italy

Thanos Myloneros, Greece

Julia Nakamura, Canada

Ndeindo Ndeikoundam Ngangro, Sweden

Rachel Neale, Australia

Lola Neufcourt, France

Roy Nielsen, Norway

Peter Nilsson, Sweden

Zary Nokhodian, Iran

Moritz Oberndorfer, Finland

Lazarus Odeny, United States

Gaëlle Oka, France

Flora Or, United States

Mark Oremus, Canada

Olof Östergren, Sweden

Snezhanka Ovcharova-Krachunova, United States

Simon Øverland, Norway

Hilal Özcebe, Turkey

Manuel Pabón-Carrasco, Spain

Joseph Palamar, United States

Nikola Panic, United States

Dimitra Panteli, Germany

M. Isabel Pasarín, Spain

Domenico Pascucci, Italy

Roberta Pastorino, Italy

Jacob Pedersen, Denmark

Sergio Perelman, Belgium

Sophie Peter, Germany

Angelo Maria Pezzullo, Italy

Sylvain Pichetti, France

Peter Piko, Hungary

Denise Pires Marafon, Italy

Olga Poluektova, Italy

Anu Polvinen, Finland

Esteban Porrini, United States

Irene Possenti, Italy

Ivan Pristas, Croatia

Rui Qi, China

Jjiabi Qin, China

Ossi Rahkonen, Finland

Rachel Rahman, United Kingdom

Svein Rasmussen, Norway

Melissa Raven, Australia

Caitlin Reid, United Kingdom

Sijmen Reijneveld, Netherlands

Wendy Rodenburg, Netherlands

Max Rohrbacher, Germany

Carlos Rojas-Roque, United Kingdom

Celine Rooze, Netherlands

Aldo Rosano, Italy

Alexandre Rossignol, United States

Olivier Rubin, Denmark

Nicolas Ruesch, Germany

Katharina Runge, Netherlands

Maria Sala, Spain

Juliana Salgado Raffaeli, Brazil

Aurelien Sallin, Switzerland

Anne-Laure Samson, United States

Romina Saucedo Araujo, Spain

Karen Saylors, United States

Mandy Schulz, Germany

Nienke Schutte, Belgium

Mathilde Sengoelge, Sweden

Sharmin Shabnam, United Kingdom

Samir Shah, United States

Orjola Shahaj, Czech Republic

Richard Shaw, United Kingdom

Mahdi Sheik, United States

Ilana Shoham-Vardi, Israel

Ruth Simmons, United Kingdom

Sandra Claire Slesinski, Germany

Anthony Staines, Ireland

Tanja Stamm, Austria

Denes Stefler, United Kingdom

Michael Stucki, Switzerland

David Stuckler, Italy

Jun Sugihara, Japan

Sakari Suominen, Finland

Viera Švihrová, Slovenia

SZYMON SZEMIK, Poland

Cristina Taddei, United Kingdom

Ilona Tamutiene, Lithuania

Judy Tan, United States

Erik Teunissen, Netherlands

Wendy Thompson, United States

Sally Thorne, Canada

Marianne Tönnessen, Norway

Liv Torheim, Norway

Nuria Torner, Spain

Perihan Torun, Turkey

Eni Tresa, Netherlands

Meltem Tuna, Canada

Olav Tveit, Norway

Trygve Ugland, Canada

Heini Vaisanen, France

Alexander Valkov, Bulgaria

Angelica Valz Gris, Italy

Paul Bastiaan van der Nat, Netherlands

Koos van der Velden, Netherlands

Christel van Dijk, Netherlands

Coen van Gool, Netherlands

Ko van Wouwe, Netherlands

Orsolya Varga, Hungary

Arturo Vargas Bustamante, United States

Alejandro Vásquez-Echeverría, United States

Veronica Velasco, Italy

bruno ventelou, France

Robert Verheij, Netherlands

Massimo Vicentini, United States

Serena Vigezzi, Denmark

Leonardo Villani, Italy

Pekka Virtanen, Finland

Chi Quynh Vo, Norway

Beate Völker, Netherlands

Daisy Volmer, Estonia

Sebastian von Schreeb, United States

Ursula Wagner, United States

Long Wang, China

Ranjit Warrier, United States

Jacques Wels, United Kingdom

Maria Wessman, Denmark

Selam Woldemariam, Austria

Biao Xu, China

Andreas Xyrichis, United Kingdom

Diego Yacamán Méndez, Sweden

Rajpal Yadav, India

Liyong Yuan, United States

Paola Zaninotto, United Kingdom

Ángel R. Zapata-Moya, Spain

Virginia Zarulli, Denmark

Chunyu Zheng, United Kingdom

Manhua Zhu, United States

Marzena Zielinska, United States

